# Contralateral orbitopterional approach for anterior skull base meningioma resection

**DOI:** 10.3171/2025.10.FOCVID25189

**Published:** 2026-01-01

**Authors:** Serdar Rahmanov, Mohammadmahdi Sabahi, Qais Alrashidi, Hamid Borghei-Razavi, Badih A. Adada

**Affiliations:** Department of Neurological Surgery, Pauline Braathen Neurological Center, Cleveland Clinic Florida, Weston, Florida

**Keywords:** anterior skull base meningioma, orbitopterional approach, contralateral approach

## Abstract

The authors present the resection of an anterior skull base parasellar meningioma causing vasogenic edema due to compression of the frontopolar vein. A contralateral orbitopterional craniotomy was chosen to provide a minimally invasive approach while avoiding morbidity from a bifrontal approach. Extradural removal of the orbital roof minimized frontal lobe retraction. The infrafrontal corridor enabled identification and preservation of the olfactory and optic nerves, the carotid artery, and the frontopolar vein. Circumferential microsurgical dissection with low-power bipolar coagulation enabled en bloc tumor removal. Postoperative MRI confirmed gross-total resection, resolution of the edema, and preservation of the vein. The patient had an uneventful recovery and intact neurological function at the 5-week follow-up.

The video can be found here: https://stream.cadmore.media/r10.3171/2025.10.FOCVID25189

## Transcript

This video demonstrates a contralateral orbitopterional approach for anterior skull base meningioma resection.

### 0:27 Clinical Presentation.

The patient is a 43-year-old right-handed female who presented with progressive frontal headaches, intermittent visual phenomena described as "spots," generalized weakness, and gait instability over the past year. She was incidentally diagnosed with a left inferior frontal lobe meningioma in January 2025 and initially managed with surveillance.

Follow-up MRI demonstrated progressive vasogenic edema with midline shift, prompting surgical intervention.

### 0:59 Neurological Examination.

On neurological examination, she was alert and fully oriented. Language, comprehension, and cranial nerves were intact. Motor examination revealed full strength in the upper extremities and mild weakness of the hip flexors bilaterally. Sensory modalities and reflexes were preserved, although gait was unsteady and required assistance.

### 1:19 Neuroimaging Findings.

Preoperative imaging revealed a left anterior skull base meningioma located adjacent to the falx and posterior to the frontal sinus. Significant vasogenic edema and midline shift were present. On contrast sequences, the lesion was seen encasing the frontopolar vein.

Given the patient’s progressive symptoms and worsening edema, microsurgical resection was planned.

### 1:47 Surgical Plan and Positioning.

Here we see the coronal MRI showing the tumor. Because of its midline and anterior location, combined with significant vasogenic edema on the left side, we selected a contralateral orbitopterional craniotomy. This approach provides a direct and minimally invasive trajectory while avoiding the morbidity of a bifrontal craniotomy.^1–3^

The orbitopterional craniotomy was designed to optimize access by removing the orbital roof extradurally, which helps minimize frontal lobe retraction. Additional exposure is obtained by sectioning the falx and drilling the crista galli.

Small craniotomies, such as the modified pterional or lateral supraorbital approaches, provide limited access to the contralateral side. These approaches increase the risk of retraction injury to the opposite frontal lobe, thereby endangering both frontal lobes.

The transeyebrow supraorbital approach was considered insufficient, as it would not provide adequate visualization across the midline.^4^

### 2:50 Positioning.

The patient was placed in the supine position, with the head elevated approximately 15° to facilitate venous drainage and rotated 30° to the left to optimize the surgical corridor.

A curvilinear skin incision was designed just behind the hairline. The temporalis muscle was then dissected in a stepwise manner, preserving the vascular supply and minimizing trauma to the muscle fibers.

A standard orbitopterional craniotomy was performed, combining the frontotemporal and orbital components in a single bone flap. This modification expands the anterior and inferior exposure, creating a wide surgical window to the anterior cranial base.

### 3:35 Operative Techniques.

Once the bone flap was removed, attention was directed to extradural work. The lateral orbital wall and sphenoid ridge were carefully drilled down, exposing the periorbita and the superior orbital fissure. This maneuver widens the operative angle to the midline and reduces the need for brain retraction.

Finally, the orbital roof was thinned in a controlled fashion and then removed extradurally. Removing the orbital roof minimizes traction on the frontal lobe, providing a flatter, more direct trajectory toward the anterior skull base lesion.

### 4:15 Opening of the Dura Mater.

Once the orbital roof had been removed, attention was turned to the dura. A careful dural incision was made, initially small, to allow controlled release of cerebrospinal fluid. CSF was first released from the epidural space and sylvian cistern, which helped reduce tension on the frontal lobe and facilitated safer intradural work. Following this, the dura was opened in a broad, curvilinear fashion.

Through the infrafrontal surgical corridor, careful dissection advanced toward the midline. The olfactory nerve was identified and protected throughout the procedure.

The optic nerve was identified. Under high magnification, the arachnoid membrane covering the nerve was carefully dissected, and the opticocarotid cistern was opened with an arachnoid knife.

The contralateral optic nerve was carefully separated from the orbital gyrus. The dissection was performed meticulously to preserve perforating vessels and surrounding vascular structures. This ensured the safe mobilization of the fronto-orbital lobe without compromising nerve function.

After extensively opening the basal cisterns, the olfactory nerve was gently dissected and mobilized from the orbital gyrus. The arachnoid membrane overlying the olfactory nerve was carefully divided with an arachnoid knife. This enabled the retraction of the orbitofrontal lobe in a methodical manner while preserving the structural integrity of the olfactory pathways.^5^

Next, the falx was coagulated and incised to expand access across the midline. This procedure exposed the crista galli, which was then carefully dissected from its dural attachments. The crista galli was drilled to widen the operative window and facilitate safe exposure and resection of the tumor.

### 6:29 Tumor Identification and Resection.

Low-setting bipolar coagulation was used throughout the procedure for hemostasis to minimize collateral thermal injury. After securing hemostasis, the tumor was carefully approached, and the frontopolar vein was identified. Notably, the tumor was found to be compressing and displacing this vein, which explains the patient’s significant vasogenic edema and midline shift seen on preoperative imaging.

After identification of the tumor and its venous relationships, resection began by detaching the tumor from its origin at the anterior skull base. This step effectively devascularized the meningioma, enabling safer removal of its highly vascularized bulk with minimal bleeding.

The tumor was then fully separated from its dural attachment, and attention was returned to the frontopolar vein. The vein was tightly adherent and partially compromised by tumor tissue. Using microsurgical techniques, the vein was gently mobilized and freed from the tumor while maintaining its patency and functional integrity.

The tumor was carefully separated from the surrounding neurovascular structures in a circumferential fashion, working exclusively within the boundaries of the natural arachnoid planes.

Low-power bipolar coagulation was selectively used to shrink tumor tissue and control small feeders without risking thermal damage to nearby eloquent structures.

As the capsule progressively loosened, the tumor began to mobilize and roll into the operative field. At this stage, the arachnoid knife was used to sharply divide the medial arachnoid attachments, completely freeing the lesion and allowing for its en bloc removal.

Hemostasis was then secured. Inspection of the tumor cavity revealed it to be clear, with no evidence of residual mass or active bleeding.

The frontopolar vein was successfully preserved. It was reinforced and protected with a thin layer of a hemostatic agent.

Attention was then turned to the tumor’s origin along the anterior skull base. Any residual dural or bony attachments were sharply dissected with microsurgical scissors. The bone at the implantation site was drilled flat to eliminate microscopic remnants and reduce the risk of recurrence.

### 9:12 Closure.

The resection cavity was irrigated with warm saline to remove debris and ensure hemostasis, with additional hemostatic material applied as needed. The dura was closed watertight, the bone flap secured with titanium plates and screws, and the wound closed in anatomical layers.

### 9:31 Outcome.

The patient was discharged on postoperative day 5 in good condition. Histopathological analysis confirmed a WHO grade I meningioma.

At 5-week follow-up, the neurological examination remained fully intact, with normal speech, preserved cranial nerves, full motor strength, intact sensation, and independent gait.

Postoperative MRI at 5 weeks demonstrated gross-total resection of the meningioma, resolution of the left frontal vasogenic edema, and preservation and normal function of the frontopolar vein.

Thank you!

## Acknowledgments

This transcript was refined using DeepL Write and Grammarly for grammar and style checks.

## Disclosures

The authors report no conflict of interest concerning the materials or methods used in this study or the findings specified in this publication.

## Author Contributions

Primary surgeon: Adada. Assistant surgeon: Alrashidi, Borghei-Razavi. Editing and drafting the video and abstract: Rahmanov, Sabahi. Critically revising the work: all authors. Reviewed submitted version of the work: Adada, Rahmanov, Sabahi, Borghei-Razavi. Approved the final version of the work on behalf of all authors: Adada. Supervision: Adada, Alrashidi, Borghei-Razavi.

## Supplemental Information

### Patient Informed Consent

The necessary patient informed consent was obtained in this study.
